# Fabrication and Effect of Strontium-Substituted Calcium Silicate/Silk Fibroin on Bone Regeneration *In Vitro* and *In Vivo*

**DOI:** 10.3389/fbioe.2022.842530

**Published:** 2022-05-13

**Authors:** Yuning Zhou, Yue Hu, Mamoru Uemura, Lunguo Xia, Xingge Yu, Yuanjin Xu

**Affiliations:** ^1^ Department of Oral Surgery, Shanghai Ninth People’s Hospital, Shanghai Jiao Tong University School of Medicine, College of Stomatology, Shanghai Jiao Tong University, Shanghai, China; ^2^ National Center for Stomatology, National Clinical Research Center for Oral Diseases, Shanghai Key Laboratory of Stomatology, Shanghai, China; ^3^ Department of Anatomy, Osaka Dental University, Hirakata, Japan; ^4^ Department of Orthodontics, Shanghai Ninth People’s Hospital, Shanghai Jiao Tong University School of Medicine, College of Stomatology, Shanghai Jiao Tong University, Shanghai, China; ^5^ Department of Oral and Cranio-Maxillofacial Science, Shanghai Ninth People’s Hospital, Shanghai Jiao Tong University School of Medicine, College of Stomatology, Shanghai Jiao Tong University, Shanghai, China

**Keywords:** strontium-substituted calcium silicate/silk fibroin, bone marrow-derived mesenchymal stem cells, osteogenesis, angiogenesis, bone regeneration

## Abstract

Achieving rapid osteogenesis and angiogenesis was the key factor for bone regeneration. In the present study, the strontium-substituted calcium silicate (SrCS)/silk fibroin (SF) composite materials have been constructed by combining the different functional component ratios of SrCS (12.5 wt%, 25 wt%) and SF. Then, the effects of SrCS/SF materials on proliferation, osteogenic differentiation, and angiogenic factor secretion of rat bone marrow-derived mesenchymal stromal cells (rBMSCs) were first evaluated *in vitro*. Moreover, the *in vivo* effect of osteogenesis was evaluated in a critical-sized rat calvarial defect model. *In vitro* studies showed that SrCS/SF significantly enhanced the cell proliferation, alkaline phosphatase (ALP) activity, and the expression of osteogenic and angiogenic factors of rBMSCs as compared with the SF and CS/SF, and the optimum proportion ratio was 25 wt%. Besides, the results also showed that CS/SF achieved enhanced effects on rBMSCs as compared with SF. The *in vivo* results showed that 25 wt% SrCS/SF could obviously promote new bone formation more than SF and CS/SF. The present study revealed that SrCS could significantly promote the osteogenic and angiogenic activities of SF, and SrCS/SF might be a good scaffold material for bone regeneration.

## 1 Introduction

In clinical practice, autologous bone transplantation, allogeneic bone transplantation, xenogeneic bone transplantation, and allogeneic material transplantation are the main methods applied for bone defect repair. However, each treatment method possesses its own advantages and disadvantages, which are unable to satisfy the needs of bone defect morphology and functional reconstruction at the same time (Wu V. et al., 2019). Therefore, bone scaffold materials have been continuously researched and developed. After implantation, bone scaffold materials can provide a three-dimensional scaffold environment, which is conducive to cell adhesion, proliferation, differentiation, and growth (Bose et al., 2017). More importantly, in addition to promoting cell colonization and osteogenic activity, it is also crucial to maintain cell viability, which depends on adequate blood supply. Previous studies discovered that after implanting *in vivo*, the survival of cells mainly depended on the degree of vascularization in the bone scaffold materials (Yan et al., 2019; Yin et al., 2019). The growth of the vascular bed around the defect could only reach the edge of the scaffold materials, while cell death could be discovered in the center, which limited the formation of new bone (Ziebart et al., 2013). In addition, the ingrowth vascular also acted as a communication network between the new bone and adjacent tissues, thus ensuring the stability of the new bone (Chim et al., 2013). Therefore, the ideal scaffold materials for bone regeneration should possess the inductive activities of osteogenesis and angiogenesis.

As a new kind of natural polymer material, silk fibroin (SF) has good biocompatibility, mechanical strength, and toughness, which attracted wide attention in the field of bone repairing biomaterials (Omenetto and Kaplan 2010; Bhattacharjee et al., 2017). However, SF is still lacking in osteoinductive activity (Zhu et al., 2011). Previous studies have shown that the compounded organic and inorganic materials could effectively enhance the biological properties of materials (Ye et al., 2011; Wang et al., 2020). Our previous research revealed that, under the osteoporotic condition, Sr-doped CS bioceramics (SrCS) could promote the osteogenic differentiation and angiogenic factor expression of rBMSCs, which could also stimulate the angiogenic activity of HUVECs (Lin et al., 2013). Moreover, it has been reported that SrCS with different concentrations could promote osteogenesis and inhibit osteoclastogenesis at the same time (Ben et al., 2020). Based on previous studies, it is expected to enhance the osteogenic and angiogenic properties of SF materials by a combination of SrCS bioceramics. However, it is still questioned whether the SrCS compound with SF materials could improve the physicochemical and biological properties of the materials simultaneously, as well as its optimum proportion ratio.

In the present study, our hypothesis is that by combining SrCS bioceramics and SF materials, novel materials (SrCS/SF) with bi-directional osteogenic/angiogenic activity could be designed. To test our hypothesis, rBMSCs were cultured on the composite materials with different concentrations of SrCS, and the effects on the proliferation, osteogenic differentiation, and secretion of angiogenic factors of rBMSCs were scientifically detected. Furthermore, the effects of the composite materials on bone regeneration were investigated in a rat critical-sized calvarial defect model *in vivo*.

## 2 Materials and Methods

### 2.1 Fabrication and Characterization of the Silk Fibroin, CS/SF, and Strontium-Substituted Calcium Silicate/SF Scaffold Materials

The CS and SrCS bioceramic ultrafine powders with 10 mol% of Ca replaced by Sr were prepared by chemical precipitation, sol–gel method, and hydrothermal reaction technology, while the SF solution was obtained by dialysis, as previously described (Ye et al., 2011; Lin et al., 2013). Then the CS or SrCS nanofibers and SF solution were mixed by ultrasonic wave. A proper amount of 400–600 μm granular NaCl particles was added (Byrne et al., 2008; Kasten et al., 2008; Lee et al., 2019), which were mixed evenly and put into the 6-well plates to a height of 8 mm. And then frozen in – 20ºC refrigerator and placed in a freeze dryer. After freeze-drying, the CS/SF and SrCS/SF scaffold materials with a large pore size of 400–600 μm were obtained. By adjusting the concentration of silk protein solution and the ratio of NaCl particles, the porosity and mechanical properties of the composite scaffolds were controlled, and finally, the composite scaffolds with 80–88% porosity were obtained. Moreover, by controlling the quality of CS or SrCS, the 12.5 wt% CS/SF, 25 wt% CS/SF, 12.5 wt% SrCS/SF, and 25 wt% SrCS/SF materials were fabricated separately. In addition, X-ray diffraction (XRD: D/max 2550V, Rigaku, Japan) and scanning electron microscopy (SEM: JSM-6700F, JEOL, Japan) have been performed to detect the characteristics, morphology, and surface topography of the materials, respectively. On the other hand, the macropore sizes of the materials were measured under electron microscopy using the direct observation from cross section method (Engin and Tas 1999). While the porosity of the materials was determined by the Archimedean method using distilled water as the determination medium (Lin et al., 2013).

The compressive mechanical properties of all scaffolds were detected using a universal mechanical testing machine (Instron, United States). The materials were stressed under the loading rate of 1 mm/min. When the compression variable reaches 60%, the stress–strain curve of the material was obtained. Then, the compression modulus at 10% deformation was calculated.

The effect of CS or SrCS addition on the degradation behavior of SF was determined by measuring their weight loss percentage in Tris-HCL buffered solution (0.1 mol/L), which is prepared as per described in the previous study (Xia et al., 2019). Then the samples were soaked in Tris-HCL buffered solution and refreshed every day. On days 1, 3, 5, 7, and 14, the samples were taken out, rinsed with deionized water, and then freeze-dried to measure the weight loss.

The 12.5 wt% and 25 wt% SrCS/SF scaffold materials were soaked in 1 ml medium without FBS and incubated for 4, 7, and 10 days. The medium was collected at each time point, and concentrations of strontium in the medium were measured by inductively coupled plasma atomic emission spectroscopy (ICP-AES; Varian, United States).

### 2.2 Isolation and Culture of Rat Bone Marrow-Derived Mesenchymal Stromal Cells

The rBMSCs were isolated and cultured following the protocols as described in the previously study (Zhou et al., 2015). Briefly, the 4-week-old male SD rats weighing 50 ± 5 g were sacrificed by overdose of pentobarbital. Then both ends of the femurs were cut off at the metaphyses, and the marrow was flushed out with 10 ml modified Eagle’s medium (MEM; Gibco, United States) supplemented with 10% fetal bovine serum (FBS; Gibco, United States) and antibiotics (penicillin 100 U/mL, streptomycin 100 U/mL). After culturing in an incubator at 37ºC with 5% CO_2_ for 4 days, the medium was first changed and then renewed every 2 days. At a confluence of approximately 90%, the rBMSCs were washed with phosphate-buffered saline (PBS) and passaged using 0.25% trypsin/ethylenediaminetetraacetic acid (trypsin/EDTA). The cells from passages 1 to 3 were used for subsequent experiments.

### 2.3 MTT Assay

To investigate the effects of different scaffold materials on cell proliferation of rBMSCs, the MTT assay was performed. First, the cells were plated on the different scaffold materials (SF, 12.5 wt% CS/SF, 25 wt% CS/SF, 12.5 wt% SrCS/SF, and 25 wt% SrCS/SF) into 96-well plates at a density of 5×10^3^ cells per piece of material, then cultured in the medium for 1, 4, and 7 days. At each time point, the materials with cells seeded were removed into other blank wells to exclude the influence from the rBMSCs adherented on the wells, and then incubated in MEM containing 10% MTT (Amresco, United States) solution at 37 °C for 4 h. Finally, DMSO was used and the absorbance of the solution was measured at 490 nm using an ELx Ultra Microplate Reader (BioTek, United States). All experiments were performed in triplicate.

### 2.4 Real-Time Quantitative PCR (RT-PCR) Analysis

To measure the expression of osteogenic and angiogenic genes of rBMSCs seeded on different materials as previously described, the RT-PCR analysis was performed at 4, 7, and 10 days. At each time point, after collecting the cells, the RNA was extracted using TRIzol reagent (Invitrogen, Carlsbad, CA, United States), and complementary DNA (cDNA) was then synthesized using a Prime-Script RT reagent kit (Takara Bio, Japan) following the manufacturer’s recommendations. Quantification for ALP, bone morphogenetic protein 2 (BMP-2), osteopontin (OPN), vascular endothelial growth factor (VEGF), and angiogenin-1 (ANG-1) were analyzed with a Bio-Rad MyiQ single-color real-time PCR system, while glyceraldehyde-3-phosphate dehydrogenase (GAPDH) was used as an internal control for normalization. All experiments were performed in triplicate.

### 2.5 Alkaline Phosphatase Activity Analysis

After the rBMSCs were seeded on the different scaffolds as described earlier, at 7 days, ALP staining was analyzed. Briefly, the cells were incubated in BCIP/NBT solution (Beyotime, Shanghai, China) in the dark at 37ºC, and the areas stained purple were regarded as positive, as the previous study described (Zhou et al., 2015). In addition, after the above experiments, SF, 25 wt% CS/SF and 25 wt% SrCS/SF groups were selected. On days 4, 7, and 10, ALP quantity analysis of BMSCs cultured on these materials was performed following the manufacturer’s instructions (Beyotime, China). First, the BMSCs were incubated with 400 μL lysis buffer at 37ºC for 4 h, and the samples were vibrated for 30 min. Then the ALP activity was quantified by absorbance at 405 nm (BioTek, United States) using p-nitrophenyl phosphate disodium (p-NPP) as the substrate and calculated according to a reference standard product. Furthermore, the total cellular protein content was measured by detecting the absorbance at 630 nm and calculating with reference to a series of BSA (Sigma, United States) standards, using the Bio-Rad protein assay kit (Bio-Rad, United States). Finally, the ALP quantitative result was accessed as pNP (mM) per milligram of total cellular proteins. All experiments were performed in triplicate.

### 2.6 Enzyme-Linked Immunosorbent Assay

To analyze the angiogenic protein expression of cells cultured on SF, 25 wt% CS/SF, and 25 wt% SrCS/SF scaffold materials, the VEGF content was measured by using a VEGF ELISA kit (Bender, United States) on days 4, 7, and 10. According to the manufacturer’s instructions, the VEGF concentration was specifically measured using a standard curve and was further normalized to the total cellular protein content, as described above. All experiments were performed in triplicate.

### 2.7 Animal Experiments

First, 9 SD rats of 8-week-old were randomly allocated into three groups: SF group, 25 wt% CS/SF group, and 25 wt% SrCS/SF group. The animals were anesthetized by intraperitoneal injection of pentobarbital (Nembutal 3.5 mg/100 g). On the scalp, a 1.0- to 1.5-cm sagittal incision was made, and the calvarium was exposed by blunt dissection. Two bilateral critical-sized defects were created by using a 5-mm diameter trephine bur (Fine Science Tools, United States). Finally, 18 critical-sized calvarial defects in 9 rats were generated and randomly filled with the scaffolds as previously described (*n* = 6), respectively. All the rats were sacrificed, and the calvarias were removed after 8 weeks.

### 2.8 Sequential Fluorescent Labeling

To investigate the new mineralized tissue at different stages, polychrome sequential fluorescent labeling was performed over a period of 8 weeks according to the method as described in the previous study (Ye et al., 2011). Briefly, the animals were intraperitoneally injected with 25 mg/kg tetracycline hydrochloride (TE, Sigma, United States), 30 mg/kg alizarin red (AL, Sigma, United States), and 20 mg/kg calcein (CA, Sigma, United States) at 2, 4, and 6 weeks after the operation, respectively.

### 2.9 Microcomputed Tomography Examination

At 8 weeks after the operation, the rats in each group were sacrificed using an overdose of pentobarbital. The calvarias were fixed in a 4% phosphate-buffered formalin solution and then detected by a microcomputed tomography (micro-CT) system (μCT-80, Scanco Medical AG, Switzerland) as described in the previous study (Zhou et al., 2017). The segmentation of bone tissue from the CS/SF and SrCS/SF was carried out by the threshold segmentation method, while the selected bone grey threshold range was 120–255. Moreover, the bone mineral density (BMD) and the trabecular thickness (Tb. Th) of the bone defects were calculated by auxiliary histomorphometric software (Scanco Medical AG, Switzerland). All experiments were performed in triplicate.

### 2.10 Histological and Histomorphometric Observation

By ascending in concentrations of alcohol ranging from 75 to 100% and embedding in polymethylmethacrylate (PMMA), the samples were dehydrated. Three longitudinal sections for each specimen were prepared as the previous study described (Zhou et al., 2017). First, the samples were observed for fluorescent labeling using CLSM (Leica TCS, Germany), and the images inside the calvarial defects were partially magnified. Then, using a personal computer-based image analysis system (Image Pro 5.0, Media Cybernetic, United States), the fluorochrome staining for new bone formation and mineralization was quantified by calculating the percentage of fluorescence area in the defect images, while the image margin was treated as the calculation range. Data pertaining to the colors yellow (TE), red (AL), and green (CA) represent bone regeneration and mineralization at weeks 2, 4, and 6 after the operation, respectively. Finally, the samples were stained with van Gieson’s (VG) picro-fuchsin for histological observation. Using Image Pro 5.0, the area of new bone formation was quantified along three randomly selected sections from the serial sections collected from each sample and reported as a percentage of the whole bone defect area. All experiments were performed in triplicate.

### 2.11 Statistical Analysis

The means and standard deviations of all data were calculated. Differences between groups were analyzed by ANOVA and the SNK post hoc or Kruskal–Wallis nonparametric procedure followed by the Mann–Whitney *U* test for multiple comparisons based on the results of the normal distribution and equal variance assumption test (Zhou et al., 2017) using SAS 8.0 software (SAS Inc., United States). A difference was considered statistically significant at a *p*-value < 0.05 (*▲ *p* < 0.05).

## 3 Results

### 3.1 Characterization of SF, CS/SF, and SrCS/SF Materials

**FIGURE 1:**
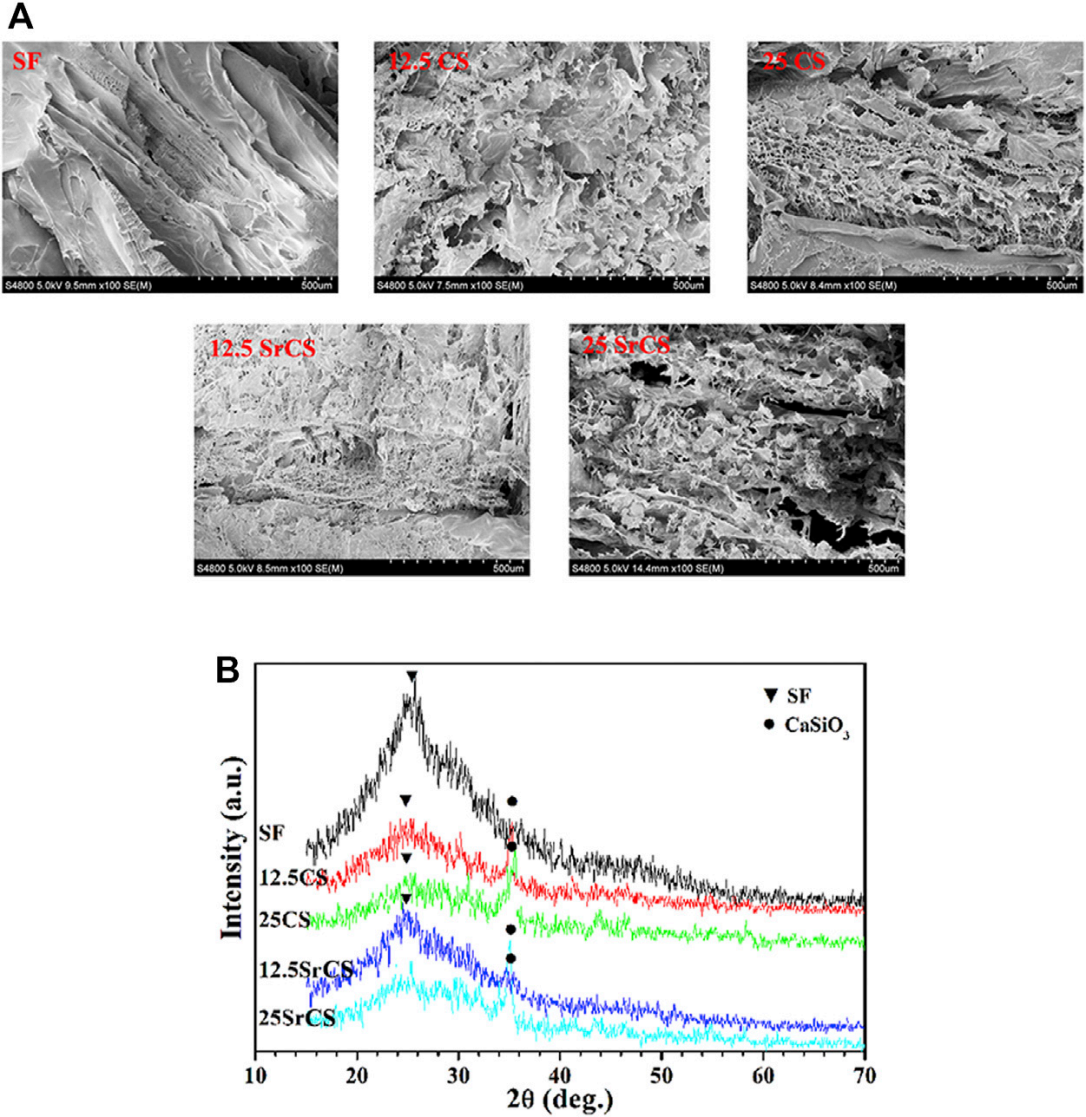
Characteristics of materials. **(A)** SEM micrographs of materials (SF: SF, 12.5 CS: 12.5 wt% CS/SF, 25 CS: 25 wt% CS/SF, 12.5 SrCS: 12.5 wt% SrCS/SF, 25 SrCS: 25 wt% SrCS/SF). **(B)** XRD patterns of materials. **(A)** Scale bar = 500 μm.

The CS/SF and SrCS/SF scaffold materials with a large pore size of 400–600 μm and 80–88% porosity were fabricated (Supplementary Table S2). As shown in SEM micrographs (Figure 1A), compared with the SF and CS/SF materials, the porosity of the SrCS/SF materials increased to a certain degree, including distributed and interconnected porosity. While the macropore sizes and porosity of SrCS/SF were in the range of 400–600 μm and 80–88%, respectively. Meanwhile, the XRD patterns (Figure 1B) showed the diffraction peaks (indicated as ▼ and ●), which suggested that both CS and SrCS could be identified as CaSiO3 phase; and confirmed that the obtained materials were compounded by CS or SrCS and SF materials, and the proportion concentration of CS or SrCS did not alter the phase composition.

**FIGURE 2:**
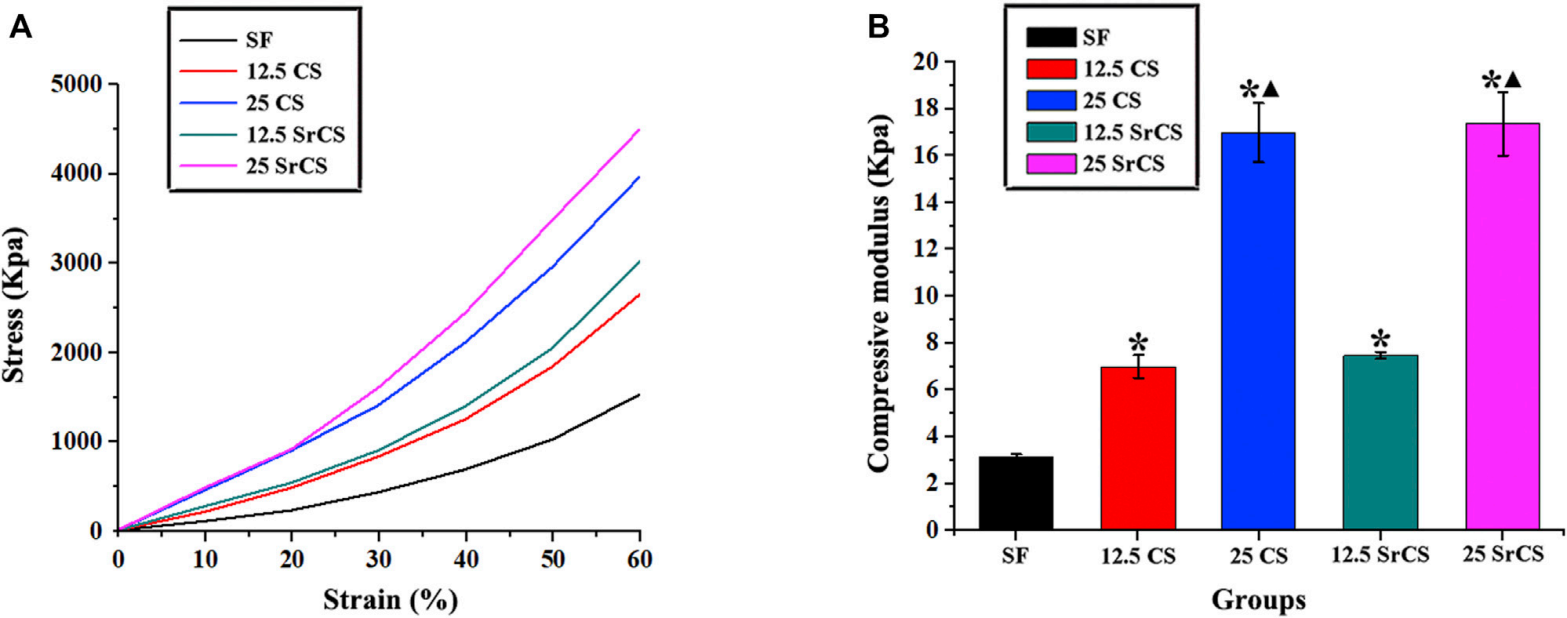
Compressive mechanical properties of materials. **(A)** Stress–strain curve of materials. **(B)** Compressive modulus of materials at 10% strain. (SF: SF, 12.5 CS: 12.5 wt% CS/SF, 25 CS: 25 wt% CS/SF, 12.5 SrCS: 12.5 wt% SrCS/SF, 25 SrCS: 25 wt% SrCS/SF). The SF group was treated as the control group. *p < 0.05 indicates the other groups vs. the SF group. ▲*p* < 0.05 indicates the 25 CS group or 25 SrCS group vs. the 12.5 CS group or 12.5 SrCS group.

The result of compressive mechanical properties of the materials showed that the structure of all materials is relatively uniform without large holes or collapses inside (Figure 2A). It can be seen that the compressive modulus of the CS/SF and SrCS/SF scaffolds was higher than that of SF, especially in the 25 wt% groups (Figure 2B). However, there was no significant difference between 25 wt% SrCS/SF scaffolds and 25 wt% CS/SF scaffolds, indicating that 25 wt% CS/SF and 25 wt% SrCS/SF scaffolds both have better mechanical properties than the other scaffolds.

**FIGURE 3:**
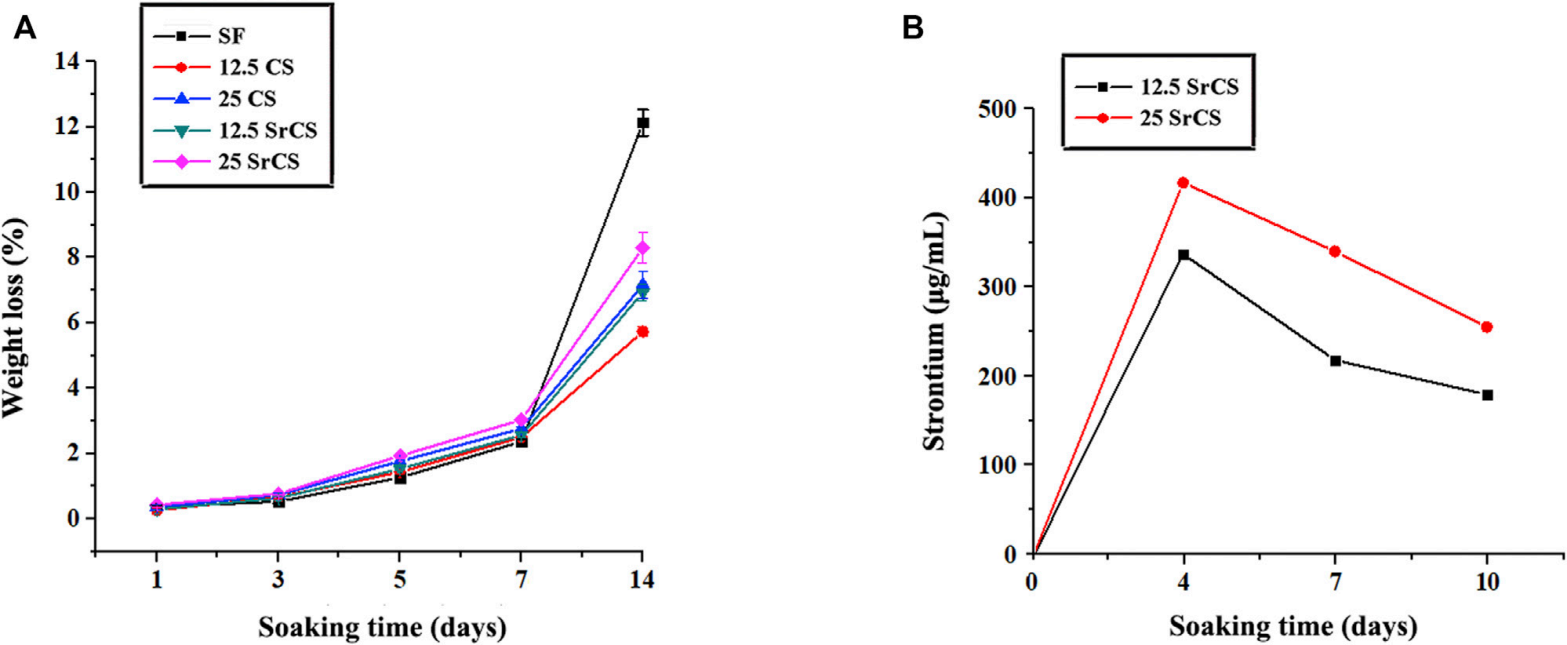
Degradation and ion release properties of materials. **(A)** Degradation behavior of materials. **(B)** Strontium release curve of 12.5 wt% and 25 wt% SrCS/SF scaffold materials. (SF: SF, 12.5 CS: 12.5 wt% CS/SF, 25 CS: 25 wt% CS/SF, 12.5 SrCS: 12.5 wt% SrCS/SF, 25 SrCS: 25 wt% SrCS/SF).

As shown in Figure 3A, the addition of CS or SrCS could reduce the degradation rate of SF, which is better cooperating with the bone regeneration rate *in vivo*. While the release curve of strontium showed that 12.5 wt% and 25 wt% SrCS/SF scaffold materials could release strontium steadily throughout the whole observation time, the concentration of released strontium became lower with time (Figure 3B).

### 3.2 MTT Analysis

**FIGURE 4:**
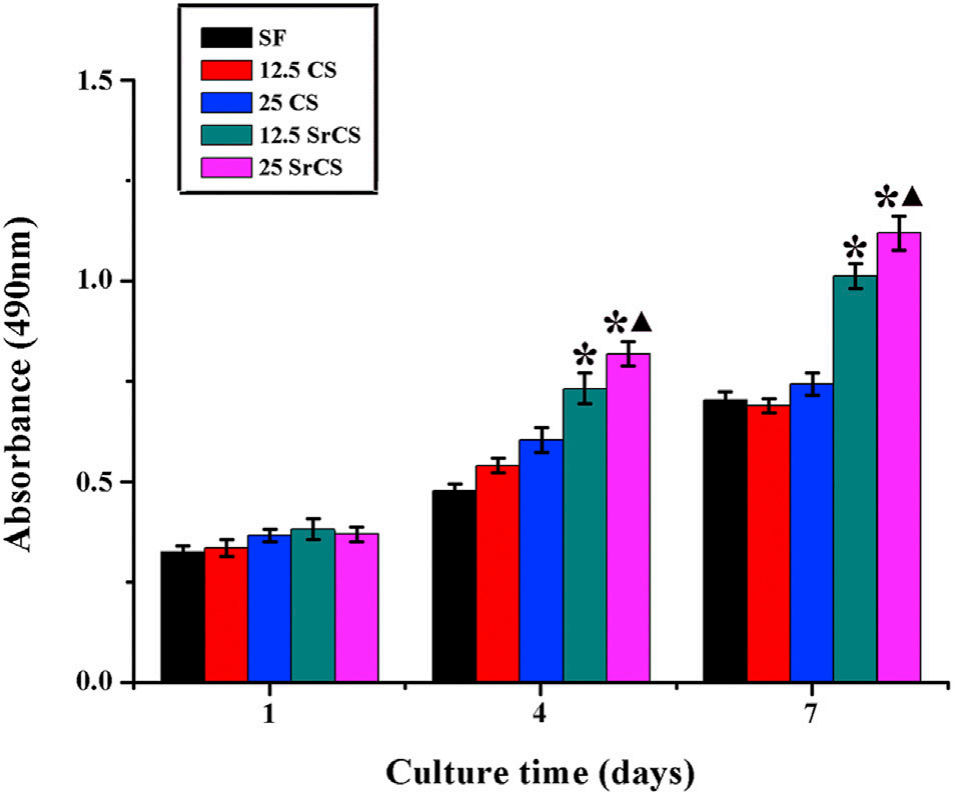
MTT assay. The effect of different materials (SF: SF, 12.5 CS: 12.5 wt% CS/SF, 25 CS: 25 wt% CS/SF, 12.5 SrCS: 12.5 wt% SrCS/SF, 25 SrCS: 25 wt% SrCS/SF) on the proliferation of rBMSCs. The SF group was treated as the control group. *p < 0.05 indicates the other groups vs. the SF group, and ▲*p* < 0.05 indicates the 25 SrCS group vs. the other groups.

To measure the proliferation of rBMSCs cultured on different materials described previously, the MTT analysis was performed on days 1, 4, and 7. In Figure 4, significantly increased cell proliferation was observed in the SrCS/SF groups than in the respective other groups on days 4 and 7. In addition, a significant difference was detected between the 25 wt% SrCS/SF group and the other groups at 4 and 7 days (*p* < 0.05).

### 3.3 RT-PCR Analysis

**FIGURE 5:**
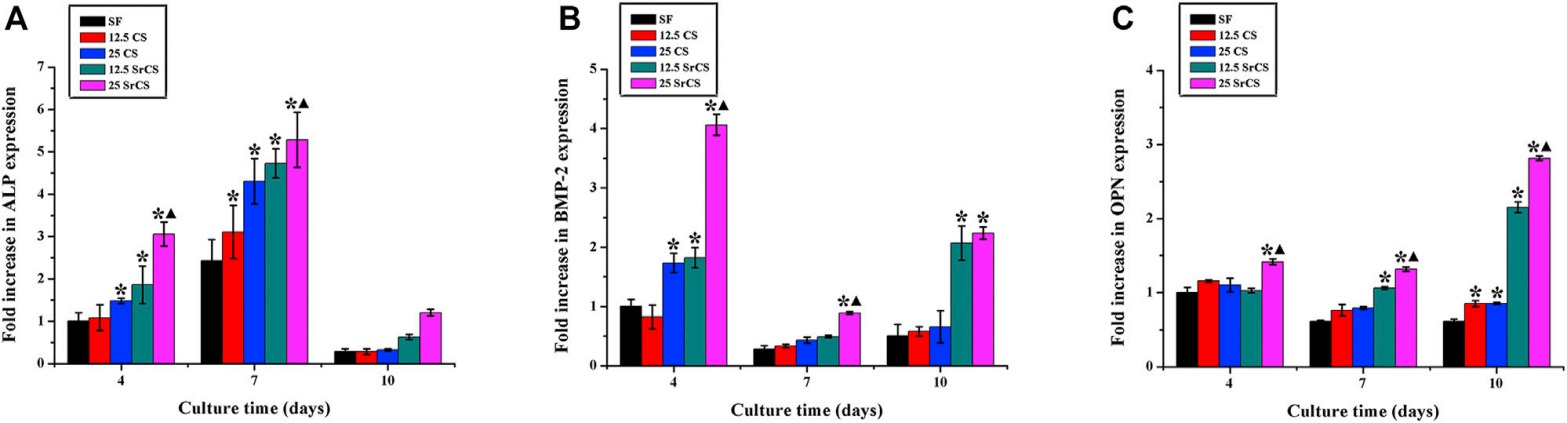
Osteogenic gene expression analysis. The osteogenic gene expression of rBMSCs seeded on different materials (SF: SF, 12.5 CS: 12.5 wt% CS/SF, 25 CS: 25 wt% CS/SF, 12.5 SrCS: 12.5 wt% SrCS/SF, 25 SrCS: 25 wt% SrCS/SF) for 4, 7, and 10 days. **(A)** ALP; **(B)** BMP-2; **(C)** OPN. The SF group was treated as the control group. *p < 0.05 indicates the other groups vs. the SF group, and ▲*p* < 0.05 indicates the 25 SrCS group vs. the other groups.

**FIGURE 6:**
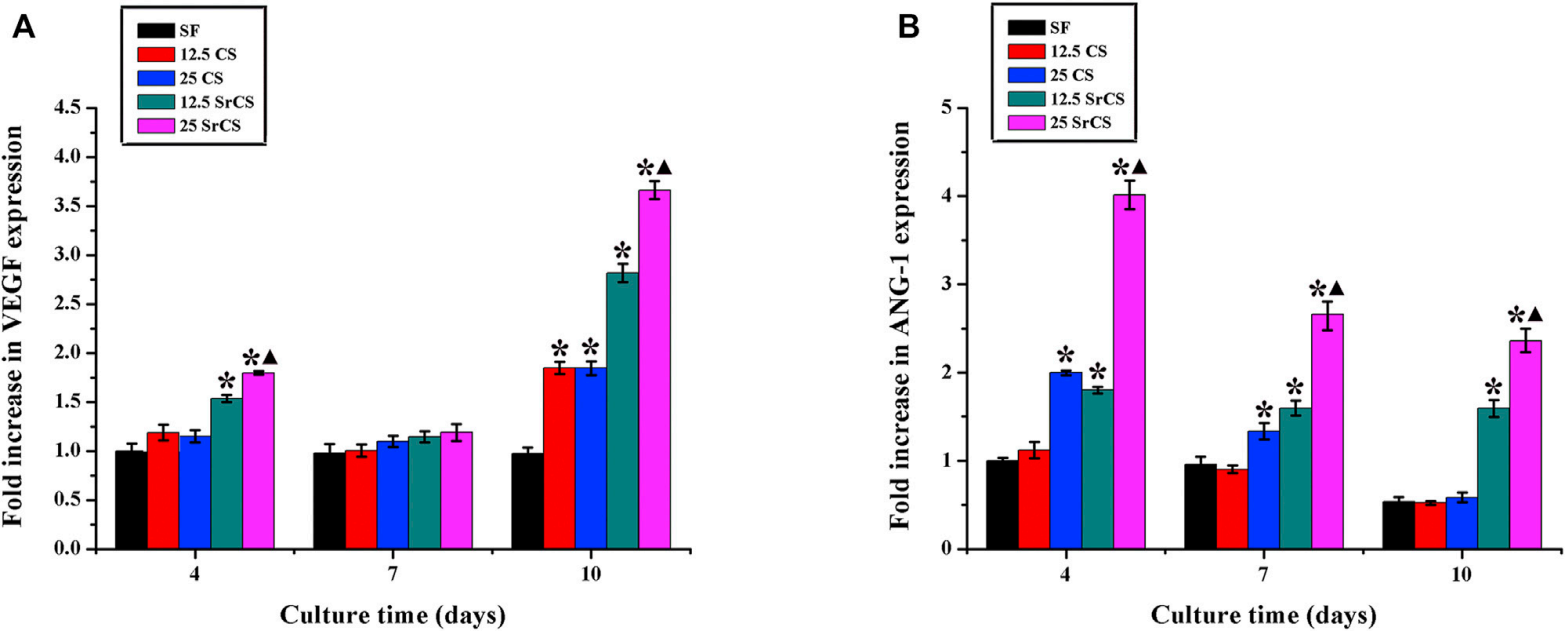
Angiogenic gene expression analysis. The angiogenic gene expression of rBMSCs seeded on different materials (SF: SF, 12.5 CS: 12.5 wt% CS/SF, 25 CS: 25 wt% CS/SF, 12.5 SrCS: 12.5 wt% SrCS/SF, 25 SrCS: 25 wt% SrCS/SF) for 4, 7, and 10 days. **(A)** VEGF; **(B)** ANG-1. The SF group was treated as the control group. *p < 0.05 indicates the other groups vs. the SF group, and ▲*p* < 0.05 indicates the 25 SrCS group vs. the other groups.

To determine the expression of the osteogenic genes, i.e., ALP, BMP-2, and OPN; and the angiogenic genes, i.e., VEGF and ANG-1 of rBMSCs seeded on different materials described above, the RT-PCR analysis was performed (Figures 5, 6). The results for osteogenic genes showed that the expression of ALP in the CS/SF and SrCS/SF groups increased significantly compared with that in the SF group, which peaked at 7 days. However, the expression of BMP-2 in the CS/SF and SrCS/SF groups was higher than that in the SF group, which peaked at 1 day and then slowed down. Additionally, the expression of OPN in the CS/SF and SrCS/SF groups increased significantly compared with that in the SF group at each time point and peaked at 10 days. On the other hand, with respect to the angiogenic genes, the expression of VEGF in the CS/SF and SrCS/SF groups was significantly higher than that in the SF group at 4 and 10 days. In addition, the expression of ANG-1 in the CS/SF and SrCS/SF groups peaked at 4 days compared with that in the SF group. More importantly, the 25 wt% was the optimum ratio.

### 3.4 Alkaline Phosphatase Activity Analysis

**FIGURE 7:**
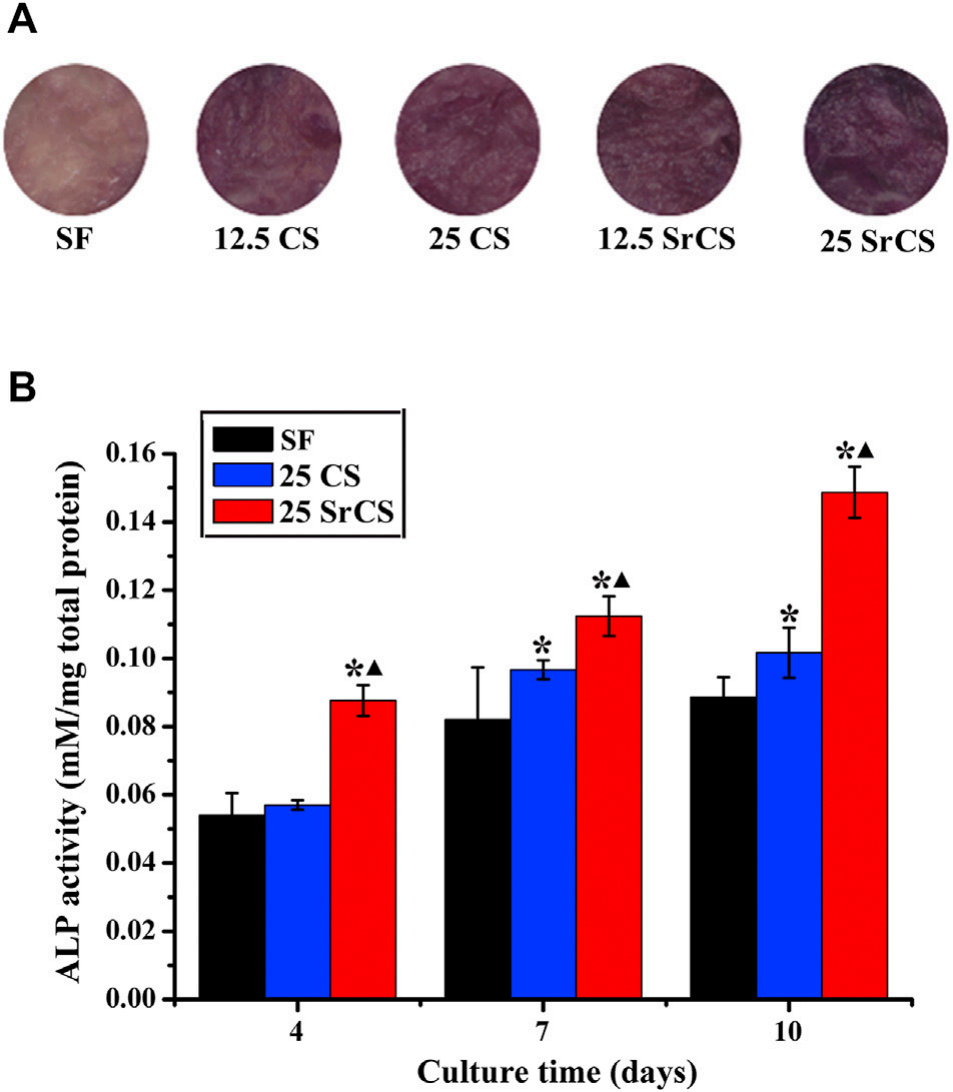
ALP activity analysis. **(A)** ALP staining of rBMSCs seeded on different materials (SF: SF, 12.5 CS: 12.5 wt% CS/SF, 25 CS: 25 wt% CS/SF, 12.5 SrCS: 12.5 wt% SrCS/SF, 25 SrCS: 25 wt% SrCS/SF) for 7 days. **(B)** ALP staining of rBMSCs seeded on different materials (SF: SF, CS/SF: 25 wt% CS/SF, SrCS/SF: 25 wt% SrCS/SF) for 4, 7, and 10 days. *p < 0.05 indicates the other groups vs. the SF group, and ▲*p* < 0.05 indicates the 25 SrCS group vs. the other groups.

To determine the early osteogenesis of rBMSCs after culturing on the different materials described previously, the ALP staining was examined. It was shown that more intense ALP staining was observed in the CS/SF and SrCS/SF groups than the SF group, especially the 25 wt% SrCS/SF group, on day 7 (*p* < 0.05, Figure 7A). As the SF, 25 wt% CS/SF, and 25 wt% SrCS/SF groups were selected, the ALP quantity analysis was detected. In Figure 7B, the result revealed that the ALP activity increased with the culture time, while the highest ALP activity was detected in the 25 wt% SrCS/SF group.

### 3.5 Vascular Endothelial Growth Factor Protein Content

**FIGURE 8:**
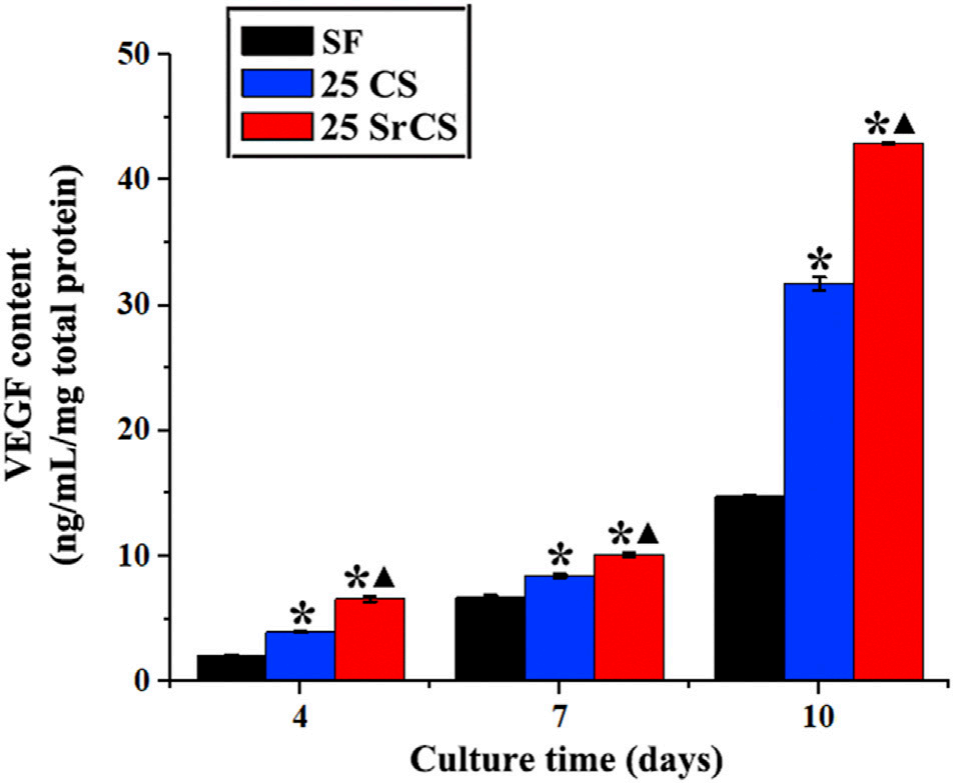
VEGF protein content test by ELISA assay. The protein level of VEGF of rBMSCs seeded on different materials (SF: SF, CS/SF: 25 wt% CS/SF, SrCS/SF: 25 wt% SrCS/SF) for 4, 7, and 10 days. *p < 0.05 indicates the other groups vs. the SF group, and ▲*p* < 0.05 indicates the 25 SrCS group vs. the other groups.

The amount of VEGF protein released from rBMSCs cultured on SF, 25 wt% CS/SF, and 25 wt% SrCS/SF scaffold materials was measured by ELISA on days 4, 7, and 10. The results showed that the VEGF protein level of 25 wt% CS/SF and 25 wt% SrCS/SF increased significantly than SF, especially in the SrCS/SF group (Figure 8).

### 3.6 Microcomputed Tomography Measurement

**FIGURE 9:**
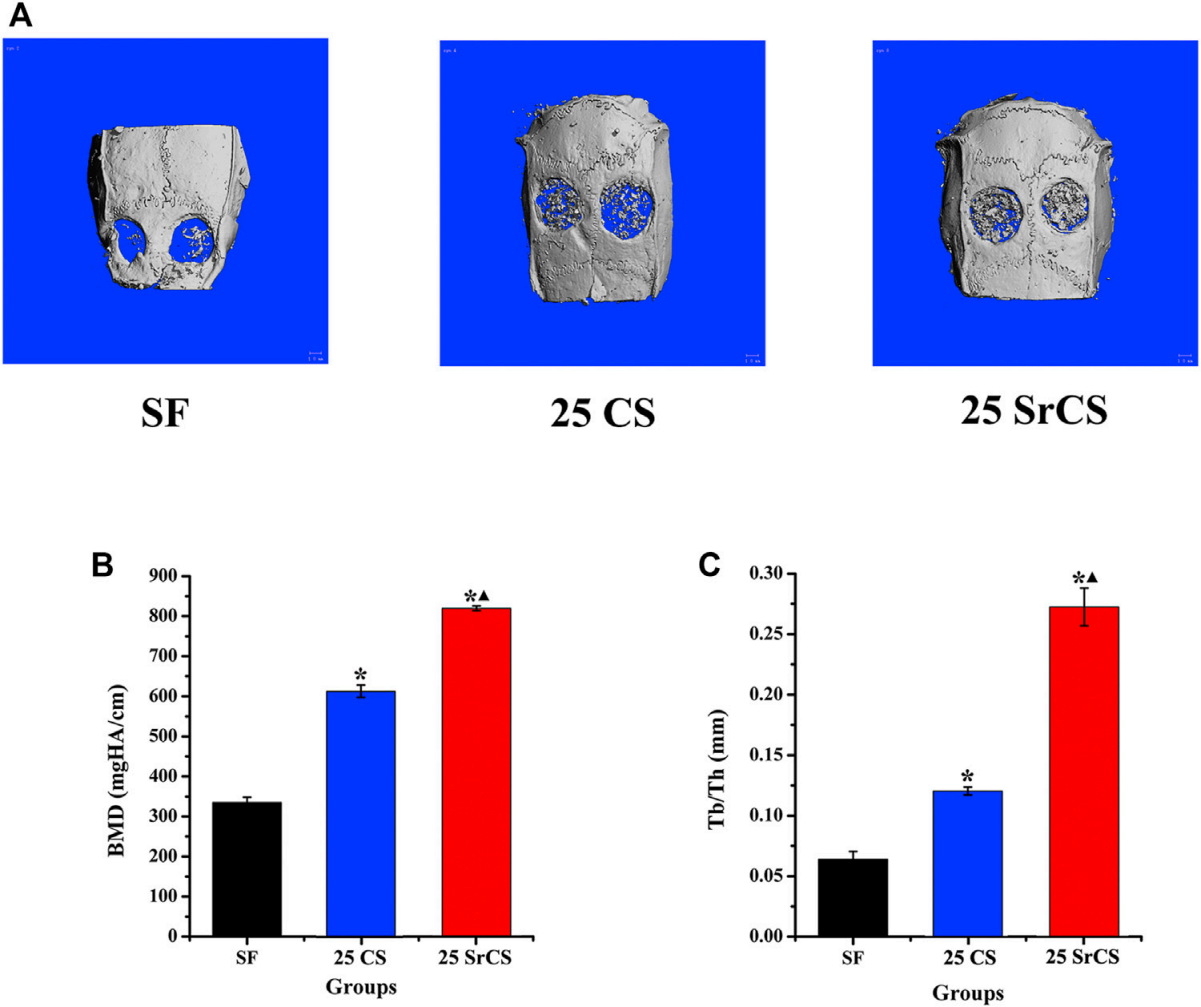
Micro-CT evaluation and morphometric analysis of calvarial bone repairing. **(A)** Representative 3D superficial image of femur bone defects (bone grey threshold range: 120-255). Morphometric analysis of bone mineral density (BMD) **(B)** and trabecular thickness (Tb. Th) **(C)** by micro-CT for each group at 8 weeks post-operation. *p < 0.05 indicates the other groups vs. the SF group, and ▲*p* < 0.05 indicates the 25 SrCS group vs. the other groups.

In Figure 9, it showed that obviously promoted new bone formation was observed in the CS/SF and SrCS/SF groups than those in the SF group, while the SrCS/SF was the most osteogenic at 8 weeks after the operation (Figure 9A). Furthermore, the similar results of BMD and Tb. Th were detected in the morphometrical analysis (*p* < 0.05) (Figures 9B,C).

### 3.7 Histological Analysis of Bone Regeneration

**FIGURE 10:**
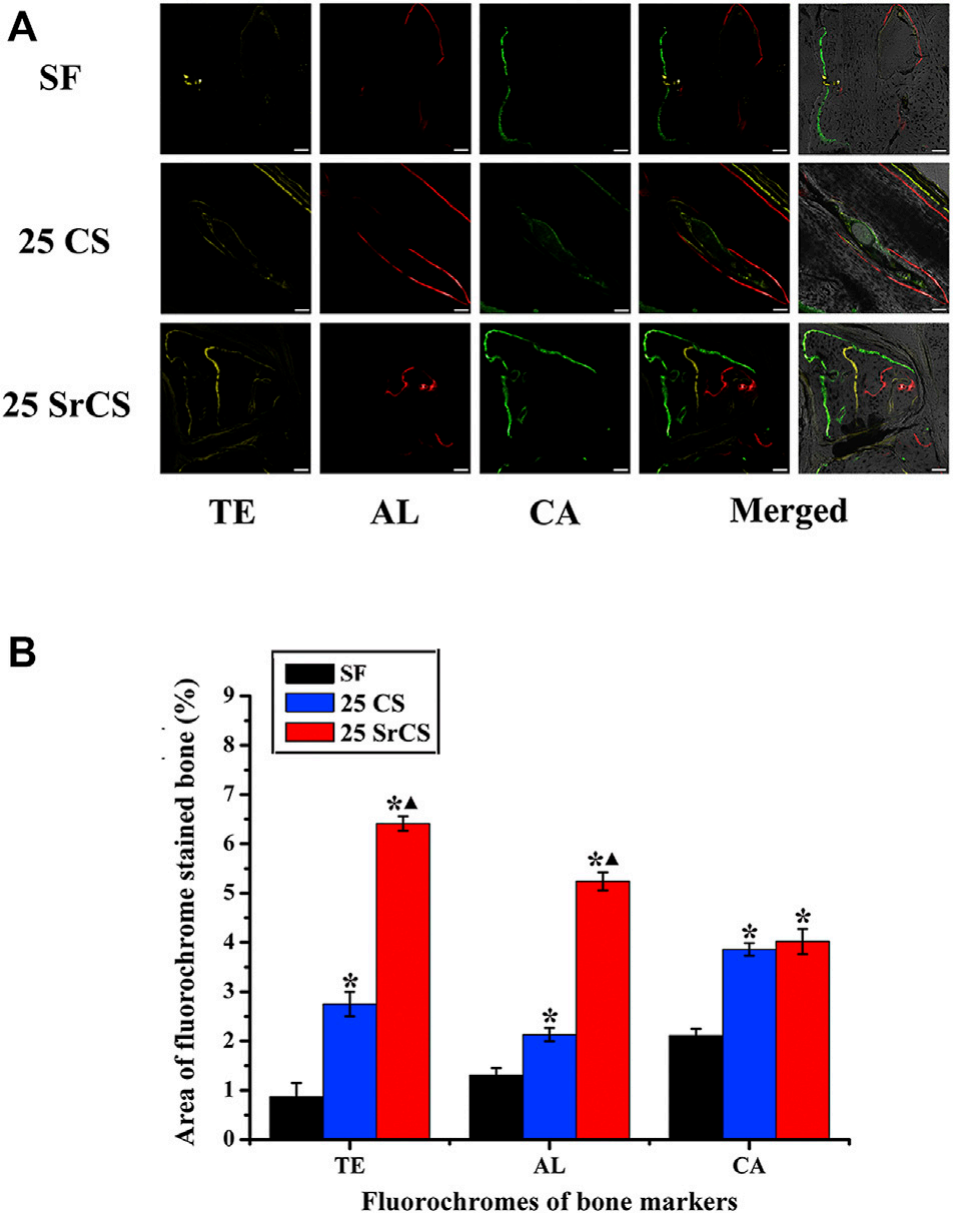
Sequential fluorescent labeling of TE, AL, and CA. **(A)** The images in yellow (TE), red (AL), and green (CA) indicate bone regeneration and mineralization at 2, 4, and 6 weeks after the operation. Merged images of the three fluorochromes or with a brightfield confocal laser microscopy image for the same group. **(B)** Percentages of TE, AL, and CA staining by histomorphometric analysis. *p < 0.05 indicates the other groups vs. the SF group, and ▲*p* < 0.05 indicates the 25 SrCS group vs. the other groups. Scale bar = 100 μm.

**FIGURE 11:**
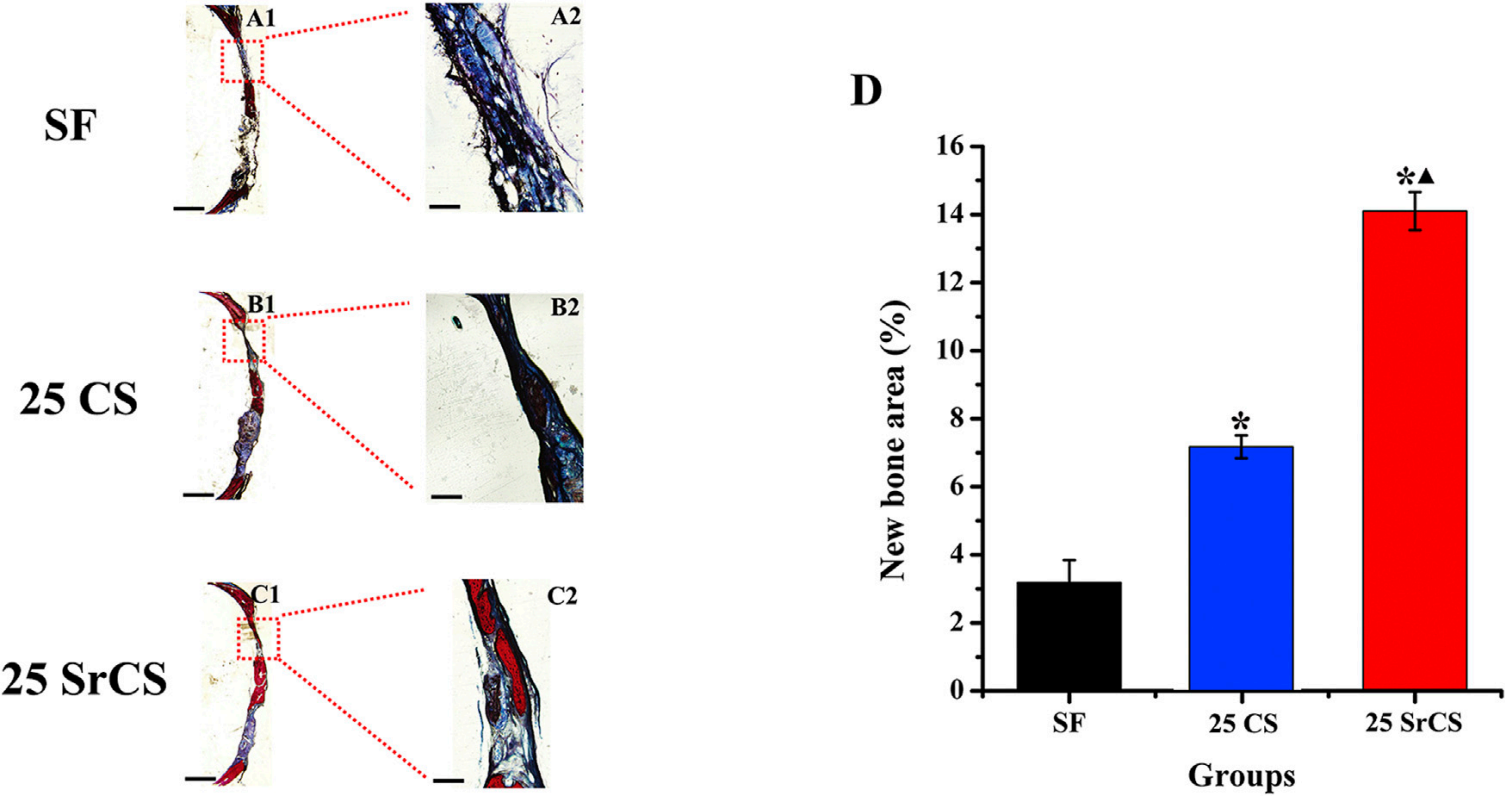
Histological images of newly formed bone in calvarial defects. **(A–C)** Histological images of newly formed bone in calvarial defects. **(D)** Percentage of the new bone area assessed by histomorphometric analysis. *p < 0.05 indicates the other groups vs. the SF group, and ▲*p* < 0.05 indicates the 25 SrCS group vs. the other groups, A1–C1: scale bar = 1 mm; A2–C2: scale bar = 100 μm.

The different fluorescent labels in Figure 10 represent new bone regeneration and mineralization at weeks 2, 4, and 6 after the operation. It revealed that, at each time, the percentages of TE labeling (yellow), AL labeling (red), and CA labeling (green) in the SrCS/SF group were significantly higher than those in the SF and CS/SF groups, while the percentages in the CS/SF group were higher than those in the SF group (*p* < 0.05). Furthermore, the results of histological analysis showed a similar conclusion The analysis of VG staining showed that more newly formed bone tissue penetrated into the defect center of the SrCS/SF group, and few new bone formations on the defect center were observed in the CS/SF group, whereas only limited new bone formation was observed on the defect bottom of the SF group (Figure 11).

## 4 Discussion

As a kind of natural high molecular fibrin, SF is an important structural protein like collagen in bone tissue. It has been confirmed that SF has many advantages, such as strong mechanical properties, long surgical application history, easy to obtain and modify, good degradability, and so on (Midha et al., 2018; Wu J. et al., 2019; Guo et al., 2021); meanwhile, the degradation products of SF have certain nutritional effects (Setzen and Williams 1997). However, it lacks sufficient osteogenic induction activity. Our previous research revealed that SrCS could promote osteogenesis and angiogenesis of osteoblasts (Lin et al., 2013). Another study also showed that SrCS could regulate the proliferation and osteogenesis of human osteoblasts (Zeng et al., 2020). Therefore, in the present study, to obtain a kind of material with good physiochemical properties and bi-directional osteogenic/angiogenic activity, it was designed by compounding SrCS and SF. As it has been found that material deposition occurs when CS or SrCS is higher than 25 wt% in the fabrication process and particle agglomeration appears in the fabricated scaffold materials, the component ratio of CS or SrCS greater than 25 wt% was not carried out in the present study.

It has been reported that the pore structure of materials could control the development of cells, which is called “contact guidance” (Kuboki et al., 1998). The pore structure of materials includes pore diameter and porosity. It has been reported that the biocompatibility of materials was mainly affected when the microstructure of materials is at the nanometer level. While the cell behavior is mainly influenced, including adhere and direction of arrangement of the cells, when the microstructure of the material is at the micron level (Cben et al., 1997). A previous study revealed that cell differentiation and proliferation could be influenced by the pore diameter of materials (Mygind et al., 2007). It has also been confirmed that high porosity could enhance the osteogenic activity of materials (Karageorgiou and Kaplan, 2005; Ardeshirylajimi et al., 2018; Lai et al., 2019). In the present study, compared with SF and CS/SF materials, the SrCS/SF materials with a large pore size of 400–600 μm and 80–88% porosity have a better pore structure. And the follow-up results showed that the SrCS/SF could promote the rBMSCs’ proliferation, osteogenesis, and secretion of angiogenic factors, as well as enhance the bone regeneration *in vivo*, which revealed that the obtained SrCS/SF materials with the appropriate pore structure have good biological activities.

As a type of widely used stem cell, BMSCs have multipotency and active proliferation, which can also be induced to secrete angiogenic factors under appropriate methods (Jiang et al., 2018). In the present study, the proliferation, osteogenesis, and secretion of angiogenic factors of rBMSCs cultured on different scaffold materials have been analyzed. It has been investigated that SrCS could promote the proliferation of osteoblast-like cells (Zeng et al., 2020; Hu et al., 2017; Chiu et al., 2019). And our previous study also showed that SrCS could promote the proliferation of rBMSCs-OVX (Lin et al., 2013). In the present study, SF and SrCS materials have been compounded with different proportion ratios. The results of the MTT assay showed that SrCS/SF could enhance the proliferation of rBMSCs rather than SF and CS/SF, which revealed that compounds with SrCS could enhance the biological properties of the proliferation of SF materials. More importantly, the results of ALP staining and RT-PCR analysis showed that, compared with SF and CS/SF materials, SrCS/SF could significantly promote the osteogenesis of rBMSCs. And the *in vivo* results also showed that the bone formation in the SrCS/SF was obviously increased than that in the SF and CS/SF. All the data revealed that rather than CS, SrCS could stimulate the osteogenic activities of SF materials. In addition, previous studies showed that the biological properties of the composite material could be affected by the proportion ratio of materials (Talal et al., 2013; Elkholy et al., 2018; Ma et al., 2018). In the present study, the materials with 12.5 wt% and 25 wt% have been fabricated, while the ratio of 25 wt% was the optimum proportion ratio both in CS/SF groups and SrCS/SF groups.

As simultaneous vascularization is a necessary condition in the process of bone regeneration, it is essential to enhance the angiogenic activity of BMSCs. It has been reported that, without modification, SF has no obvious angiogenic properties neither *in vitro* nor *in vivo* (Bai et al., 2011; Sun et al., 2016). Otherwise, our previous studies investigated that CS and SrCS could induce angiogenesis of BMSCs and HUVECs to some extent (Lin et al., 2013; Wang et al., 2020). In the present study, the results of RT-PCR analysis revealed that SrCS/SF could significantly promote the expression of angiogenic factors of rBMSCs more than SF and CS/SF. And the 25 wt% also was the optimum proportion ratio. All the data revealed that rather than CS, SrCS could upregulate the osteogenic but also angiogenic activities of SF materials, especially for the ratio of 25 wt%.

## 5 Conclusion

In conclusion, compared with SF and CS/SF, SrCS/SF could obviously enhance the cell proliferation, osteogenic differentiation, and angiogenic factor expression of rBMSCs, and the optimum ratio was 25 wt%. Furthermore, the 25 wt% SrCS/SF could promote osteogenesis *in vivo* more than SF and 25 wt% CS/SF. It is suggested that SrCS/SF with bi-directional osteogenic/angiogenic activity may be a good scaffold material for bone regeneration.

## Data Availability

The original contributions presented in the study are included in the article/Supplementary Material, further inquiries can be directed to the corresponding authors.
