# Supplementary material for: Fabrication and Effect of Strontium-Substituted Calcium Silicate/Silk Fibroin on Bone Regeneration In Vitro and In Vivo
Source: Front Bioeng Biotechnol. 2022 May 13;10:842530. doi: 10.3389/fbioe.2022.842530 (PMC9136068; doi:10.3389/fbioe.2022.842530)
Supplement: Supplementary file 1 [file Table1.DOC]

**Table S1. The primer sequences for real-time quantitative RT-PCR.**

| Gene | Forward (5'-3') | Reverse (5'-3') |
| --- | --- | --- |
| ALP | ACTGTGGACTACCTCTTG | GGTCAGTGATGTTGTTCC |
| BMP-2 | TGGGTTTGTGGTGGAAGTGGC | TGGATGTCCTTTACCGTCGTG |
| OPN | CCAAGCGTGGAAACACACAGCC | GGCTTTGGAACTCGCCTGACTG |
| VEGF | GGCTCTGAAACCATGAACTTTCT | GCAATAGCTGCGCTGGTAGAC |
| ANG-1 | GGACAGCAGGCAAACAGAGCAGC | CCACAGGCATCAAACCACCAACC |
| GAPDH | TTCGACAGTCAGCCGVATCTT | ATCCGTTGACTCCGACCTTCA |
