# Supplementary material for: Fabrication and Effect of Strontium-Substituted Calcium Silicate/Silk Fibroin on Bone Regeneration In Vitro and In Vivo
Source: Front Bioeng Biotechnol. 2022 May 13;10:842530. doi: 10.3389/fbioe.2022.842530 (PMC9136068; doi:10.3389/fbioe.2022.842530)
Supplement: Supplementary file 2 [file Table2.DOC]

**Table S2. Large pore size and porosity data of materials.**

| Group | Large pore size (μm) | Porosity (%) |
| --- | --- | --- |
| 12.5 CS | 492.4 | 86.0 |
| 25 CS | 584.5 | 83.4 |
| 12.5 SrCS | 508.3 | 87.8 |
| 25 SrCS | 563.2 | 87.0 |
